# Human iPSC cardiomyocyte patch transplantation modifies extracellular matrix and fibroblast behavior after myocardial infarction

**DOI:** 10.1016/j.isci.2026.115341

**Published:** 2026-03-11

**Authors:** Kosuke Torigata, Ryohei Matsuura, Fumiya Nagatomo, Moe Thiha, Takao Hikita, Hiroko Iseoka, Hiromitsu Takagi, Uichi Koshimizu, Hiroki Sakakima, Satoshi Izumi, Asuka Hatano, Thomas Braun, Yoshiki Sawa, Shigeru Miyagawa, Masanori Nakayama

**Affiliations:** 1 Cuorips Inc., Tokyo, Japan; 2 Daiichi Sankyo Co., Ltd., Tokyo, Japan; 3 Max Planck Institute for Heart and Lung Research, Laboratory for Cell Polarity and Organogenesis, Bad Nauheim, Germany; 4 Department of Cardiovascular Surgery, Osaka University Graduate School of Medicine, Osaka, Japan; 5 Department of Mechanical Engineering, School of Engineering, The University of Tokyo, Tokyo, Japan; 6 Department of Pathophysiology and Drug Discovery, Graduate School of Medicine, Dentistry and Pharmaceutical Sciences Okayama University, Okayama, Japan; 7 MaxPlanck Institute for Heart and Lung Research, Department of Cardiac Development and Remodeling, Bad Nauheim, Germany

**Keywords:** cell biology, fibrosis, stem cell research

## Abstract

Myocardial infarction (MI) followed by chronic heart failure is the main cause of mortality of heart diseases. Although reparative cell transplantation therapies with pluripotent stem cell-derived cardiomyocytes (CMs) represent a promising therapeutic strategy, molecular mechanisms of the therapy remain elusive. Here, we show that transplantation of the human induced pluripotent stem cell (hiPSC)-derived CM patch onto the damaged heart after MI increases the ratio of collagen type I against collagen type III to modulate alignment of the collagen fibers at the infarcted zone. As a result, tissue elasticity of the heart is improved, and fibrosis at the remote zone is reduced. Mechanistically, we find that hiPSC-derived CM patches secrete TGF-β1, directly inducing collagen type I production in fibroblasts but not collagen type III. Our results suggest the direct effect of the transplanted CM patch on the cardiac fibroblasts to improve elasticity of the damaged heart, resulting in functional recovery after MI.

## Highlights

• **hiPSC cardiomyocyte patches increase collagen I/III ratio after myocardial infarction**
• Collagen remodeling improves fiber alignment and enhances cardiac tissue elasticity
• CM patches secrete TGF-β1 to induce collagen type I in cardiac fibroblasts
• Fibroblast modulation reduces remote fibrosis and improves cardiac function after MI

## Graphical abstract

**Figure:**
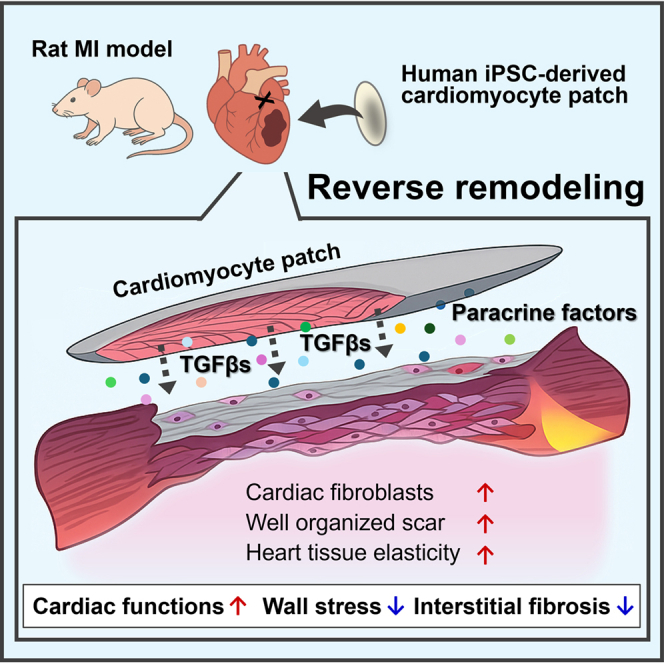

## Teaser

Cell biology; Fibrosis; Stem cells research

## Introduction

Myocardial infarction (MI) results in significant morbidity and disability and is one of the leading causes of death worldwide.^1^ While early reperfusion is clinically effective and is the key strategy for the clinical management of MI, restoration of blood flow to the ischemic tissue induces cell death and loss of myocardial and coronary function. To recover damaged heart tissues, stem cell-derived regenerative therapies, including stem cell transplantation and stem cell-derived cardiomyocyte (CM) transplantation, have been developed.^2^^,^^3^

Cardiac progenitor cells (CPCs) are thought to be multipotent cells residing in the adult heart tissues, self-renewing and generating coronary vessels and CMs.^4^ Cardiac stem cells could contribute to the regeneration of CM formation following experimental MI in mice.^5^ Various studies of heart stem cell transplantation following MI have shown an improvement of cardiac function; meanwhile, the frequency of stem cell engraftment and number of newly generated CMs appear too low to explain the described heart functional recovery.^6^^,^^7^ Accordingly, transplanted stem cells, such as CPCs or fractionated bone marrow mononuclear cells (MNCs), are believed to secrete paracrine factors including vascular endothelial growth factors (VEGFs), fibroblast growth factor 2 (FGF2), and hepatocyte growth factor (HGF). These secreted factors are thought to promote angiogenesis, neovascularization, and cell survival.^8^^,^^9^ Human mesenchymal stem cells (hMSCs) are also shown to affect matrix metalloproteinase (MMP) synthesis in cardiac fibroblasts.^10^^,^^11^ In contrast, a recent report suggests that transplantation of MNCs and CPCs expressing receptor tyrosine kinase KIT improves heart function through an acute sterile immune response characterized by the temporal and regional accumulation of inflammatory macrophages.^12^ This macrophage response alters the activity of cardiac fibroblasts, reduces the extracellular matrix content in the border zone, and enhances elasticity of the injured area. Furthermore, injection of the inflammatory mediator zymosan results in improved cardiac function.^12^ These observations suggest that the functional benefit of the adult stem cell transplantation may be based on an acute inflammatory-based wound healing process that rejuvenates the infarcted area of the heart after MI.

CMs derived from embryonic stem cells (ESCs) and induced pluripotent stem cells (iPSCs) have shown to share characteristics and functional properties of the heart tissue,^13^ thereby being potential sources of therapeutic CMs. Several studies have shown that a fraction of the stem cell-derived CMs successfully engrafts and couples with host myocardium in a synchronized manner to improve cardiac function.^14^ Allogenic transplantation of iPSC-derived CMs allows graft survival for 12 weeks without chronic rejection.^15^ A recent study using an optogenetic approach shows that transplanted CMs actively support the contractile heart function.^16^ Moreover, the paracrine effect of the transplanted CMs is suggested to induce angiogenesis.^17^ However, the mode of action of the stem cell-derived CM transplantation on the improved heart function remains elusive.

In line with these observations, we transplanted the human iPSC (hiPSC)-derived CM patch onto an immunodeficient rat MI model. Then, functional recovery of the hiPSC-derived CM patch-transplanted heart was confirmed. Importantly, we found that the production of collagen type I was increased in the infarct zone (IZ) but not that of collagen type III. Alignment of the collagen fibers in the IZ was improved, suggesting the altered character of the material properties of the damaged region. Interestingly, computational modeling revealed that characteristic alteration of the IZ decreased the average strain in the remote zone (RZ) in the heart. Consistent with this, interstitial fibrosis was improved in the RZ of the hiPSC-derived CM patch-transplanted heart. The transcriptome analysis of the hiPSC-derived CMs and the host heart cells revealed that TGF-β secretion was observed from the hiPSC-derived CMs *in vitro* and *in vivo*. Moreover, co-culture of the hiPSC-derived CMs with the cardiac fibroblasts increased collagen type I expression, which was suppressed by treatment with a TFG-β inhibitor. Conversely, collagen type III expression was not induced by co-culture of cardiac fibroblasts with hiPSC-derived CMs. Our results suggest that the paracrine effect of the hiPSC-derived CM patch might improve elasticity of the damaged heart after MI via directly affecting collagen type I production in the cardiac fibroblasts.

## Results

### hiPSC-derived CM patch engrafted, aligned, and survived on the infarcted immunodeficient rat heart 12 weeks after transplantation

**Figure 1:**
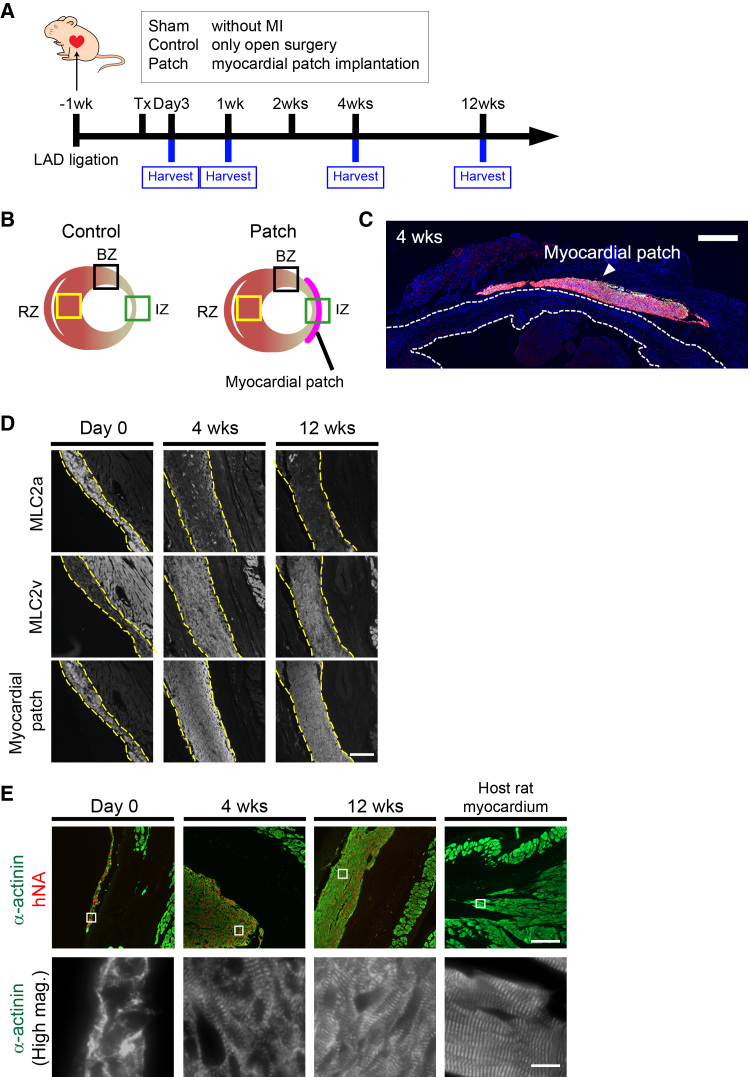
Transplanted hiPSC-derived CM patch engrafted, survived, and aligned in the immunodeficient rat MI model for 12 weeks (A) Experimental scheme using immunodeficient rats (F344/NJcl-rnu/rnu). (B) Diagram of the heart transplanted with the hiPSC-derived CM patch. IZ, infarct zone; BZ, border zone; RZ, remote zone. (C) Engrafted hiPSC-derived CM patch 4 weeks after transplantation is shown. hcTnT, red; human nuclei, green; DAPI, blue. Scale bar, 1 mm. (D) Maturation of the hiPSC-derived CM patch after transplantation. Immunoreactive signal against anti-MLC2a and anti-MLC2v antibodies is shown. The transplanted CMs are shown with immunostaining using an anti-hcTnT antibody or staining with MitoTracker and indicated with yellow dot lines. Scale bar, 200 μm. (E) Sarcomere development of the transplanted hiPSC-derived CM patch. hNA, red; α-actinin, green. Enlarged images of indicated region in the upper part are shown in the lower part. Scale bars, 200 μm or 12.5 μm.

To gain further insight into the role of transplantation of stem cell-derived CMs on cardiac functional recovery, we applied a CM patch differentiated from hiPSCs to an immunodeficient rat MI model in the subacute phase. MI was induced by ligating the proximal left anterior descending coronary artery (LAD) in 6- to 8-week-old male nude rats. One week after LAD ligation, the hiPSC-derived CM patches were transplanted onto the surface of the IZ of the rat hearts (Figures 1A and 1B). Three days, 1 week, 4 weeks, or 12 weeks after transplantation, those rats were scarified for further analysis (Figure 1A). Engrafted hiPSC-derived CM patches visualized with human cardiac troponin T (hcTnT) were confirmed 4 weeks after transplantation (Figure 1C). Cardiac myosin consists of two isoforms of myosin light chain 2, MLC2a and MLC2v. MLC2v expression is known as a marker of CM maturity, while MLC2a is considered a marker of immature CMs.^18^ Immunoreactive signals corresponding to an anti-MLC2a antibody and an anti-MLC2v antibody were confirmed shortly after transplantation in the hiPSC-derived CM patches (Figure 1D). At 4 and 12 weeks after transplantation, the immunoreactivity against the anti-MLC2a antibody decreased, whereas that against the anti-MLC2v antibody increased (Figure 1D). In addition, sarcomeres in the transplanted patch developed over time from day 0 to 12 weeks (Figure 1E). These observations suggest that the transplanted hiPSC-derived CM patch successfully grafted, aligned, and survived on the immunodeficient heart for at least 12 weeks.

### hiPSC-derived CM patch-transplanted heart showed functional recovery after MI

**Figure 2:**
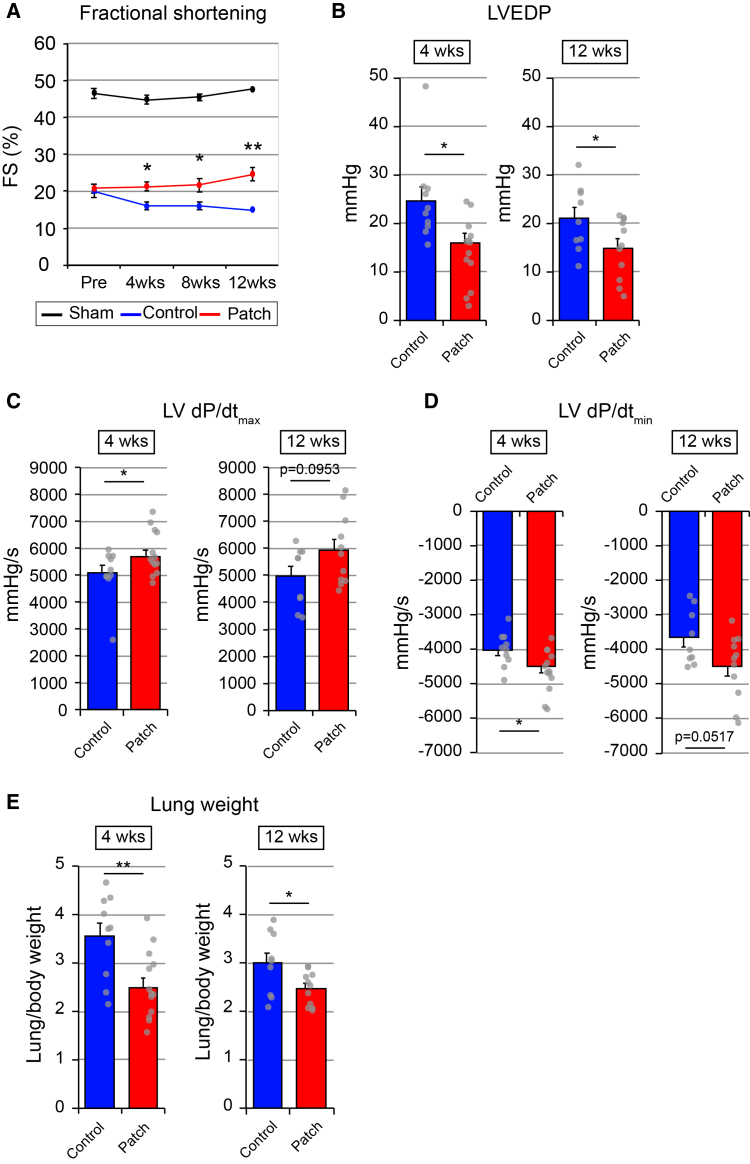
Functional recovery of the heart after hiPSC-derived CM patch transplantation (A) Untransplanted sham control (*N* = 9) and hiPSC-derived CM patch transplanted rats (*N* = 10) following MI, and sham control (*N* = 5) at the indicated time after transplantation. Repeated-measures ANOVA revealed a trend for a group effect (*p* = 0.054), and post hoc comparisons at each time point with Bonferroni correction are shown. ∗*p* < 0.05, ∗∗*p* < 0.01. Data represent mean ± SEM. (B–D) Analysis of LVEDP, LV dP/dt max, and LV dP/dt min in untransplanted control (4 weeks, *N* = 11; 12 weeks, *N* = 7) and hiPSC-derived CM patch-transplanted rats (4 weeks, *N* = 13; 12 weeks, *N* = 10) following MI at the indicated time after transplantation. Two-tailed unpaired *t* test, ∗*p* < 0.05. Data represent mean ± SEM. (E) Lung weight of untransplanted control (4 weeks, *N* = 10; 12 weeks, *N* = 7) and hiPSC-derived CM patch-transplanted rats (4 weeks, *N* = 13; 12 weeks, *N* = 10) following MI at the indicated time after transplantation. Two-tailed unpaired *t* test, ∗*p* < 0.05, ∗∗*p* < 0.01. Data represent mean ± SEM.

Next, we examined the effect of the transplanted hiPSC-derived CM patch on functional recovery of the damaged heart. To evaluate maladaptive left ventricle (LV) remodeling, echocardiography was carried out. LV fractional shortening (FS) was decreased after MI compared to the sham control, which was significantly improved in the hiPSC-derived CM patch-transplanted group at 4, 8, and 12 weeks after transplantation (Figure 2A). To examine intraventricular pressure at LV, left ventricular end-diastolic pressure (LVEDP) for diastolic function was measured using the catheter at 4 and 12 weeks after transplantation (Figure 2B). LVEDP in the patch-transplanted group was significantly lower than that in control at 4 and 12 weeks after transplantation (Figure 2B). To measure intraventricular pressure in LV, the maximal rate of pressure changes during systole (dP/dt max) for a steady-state index of systolic function (Figure 2C) and the minimum rate of pressure decay (dP/dt min) for a relaxation index (Figure 2D) were measured using the catheter at 4 and 12 weeks after transplantation. LV dP/dt max and dP/dt min were significantly recovered in the patch-transplanted group compared to control animals after 4 and 12 weeks (Figures 2C and 2D). Moreover, lung weight, which can indicate congestive heart failure, was significantly lower in the patch-transplanted groups than that in control (Figure 2E). Consistent with previous reports,^14^^,^^19^ these results indicate functional recovery of the heart after the hiPSC-derived CM patch transplantation.

### Wall thickness including the hiPSC-derived CM patch did not correlate with recovered function

Transplanted stem cell-derived CMs may be expected to increase wall thickness, thereby improving contractility of failing heart via myocardial remuscularization or regeneration. However, we did not observe any clear morphological improvement or incorporation of hiPSC-derived cells into the scar region (Figures 1C and S2A). The scar size and wall thickness of the host heart were comparable between control and the patch-transplanted groups (Figures S2B and S2C). When the thickness of the patch was included, LV wall thickness of the transplanted group tended to be more than that of control (Figure S2C). Although functional recovery of the heart was confirmed at 4 and 12 weeks after transplantation, the value of fractional shortening and LVEDP of individual rats at 12 weeks after transplantation did not correlate with wall thickness including the patch (Figures S2D and S2E).

### hiPSC-derived CM patch transplantation altered directional organization of collagen fibers

**Figure 3:**
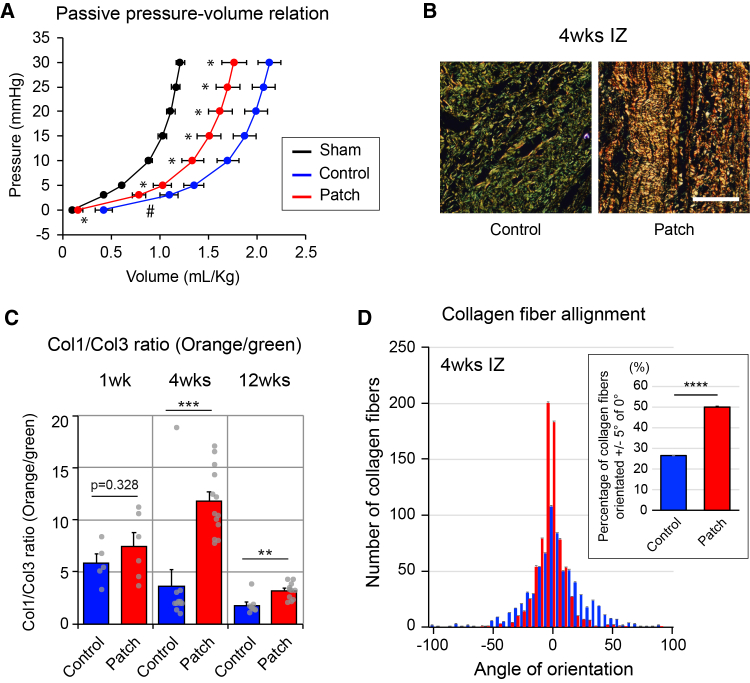
hiPSC-derived CM patch transplantation promoted collagen fiber alignment (A) Ventricular passive pressure-volume relationship of untransplanted control (*N* = 10) and hiPSC-derived CM patch-transplanted rats (*N* = 13) following MI at 4 weeks after transplantation is shown. #*p* < 0.1, ∗*p* < 0.05. Data represent mean ± SEM. two-tailed unpaired *t* test. *p* values adjusted for multiple testing using the Bonferroni method. (B) Visualization of collagen fibers in the infarct zone using a linear-polarizing microscopy. Samples were examined by Pico-Sirius Red staining. The signal corresponding to the collagen type I fiber is shown in orange, while that corresponding to collagen type III is shown in green. The samples were collected from the rats 4 weeks after transplantation. Scale bar, 200 μm. (C) Quantification of the ratio between collagen type I and collagen type III expression at 1 week (*N* = 5, control; *N* = 6, patch), 4 weeks (*N* = 11, control; *N* = 13, patch), and 12 weeks (*N* = 9, control; *N* = 11, patch) after transplantation. Two-tailed unpaired *t* test, ∗∗*p* < 0.01, ∗∗∗*p* < 0.001. Data represent mean ± SEM. (D) Quantification of collagen fiber alignment. Number of collagen fibers within a given angle orientation is represented with the maximum normalized to 0 ± 5°, *N* = 11 control, *N* = 13 transplanted rats. The angle of fibers more than 100 per image from each animal was measured. At least, 5 images were obtained for calculation from each animal. Data represent mean ± SEM, two-tailed unpaired *t* test, ∗∗∗∗*p* < 0.0001.

A previous report suggests the improved elasticity after stem cell transplantation as a consequence of inflammation via modulating collagen production.^12^ To investigate the effect of the hiPSC-derived CM patch transplantation on elasticity of the heart, we carried out left ventricular end-diastolic pressure-volume relationships in the perfused hearts from the sham-operated, post-MI with or without the patch-transplanted rats. After MI, end-diastolic pressure-volume relationship was increased, which showed a marked leftward/upward shift in the patch-transplanted group (Figure 3A), suggesting improved elasticity of the heart.

Collagen type III and type I are abundantly expressed in the cardiac tissue.^20^ Dynamic changes of their expression levels and patterns are observed during a wound healing process of the cardiac tissue, which is important for scar formation and maturation.^21^ Collagen type III is produced in greater amounts during early extracellular matrix (ECM) formation but is gradually degraded and replaced by collagen type I to increase the strength of the repair, which is further enhanced by collagen cross-linking. The organization of collagen fibers in the scar is important for the anisotropic mechanical properties of the scar.^21^ To examine the effect of the hiPSC-derived CM patch on ECM formation on the scar, we performed native PAGE followed by western blotting analysis with rat heart lysates from the IZ. Collagen type III alpha 1 chain (COL3A1) expression was higher in the control animals than that in the hiPSC-derived CM patch-transplanted group, whereas collagen type I alpha 1 chain (COL1A1) expression was lower in the control animals at 4 weeks after transplantation (Figures S3A and S3B). The collagen content and alignment of the IZ were examined using a linear-polarizing microscopy. Consistent with western blotting, the ratio of collagen type I/collagen type III was higher in the hiPSC-derived CM patch-transplanted animals than the control animals (Figure 3B). A significant difference between the two groups was confirmed at 12 weeks after transplantation, though the difference was smaller than that after 4 weeks (Figures 3B and 3C). Even in animals transplanted with the hiPSC-derived CM patch, after a week from transplantation, we could observe a tendency for higher expression of collagen type I than collagen type III (Figure 3C). Importantly, the hiPSC-derived CM patch transplantation resulted in directional organization of collagen fibers, suggesting altered material properties of the damaged tissue (Figure 3D).

### hiPSC-derived CM patch transplantation improved elasticity of the heart after MI

**Figure 4:**
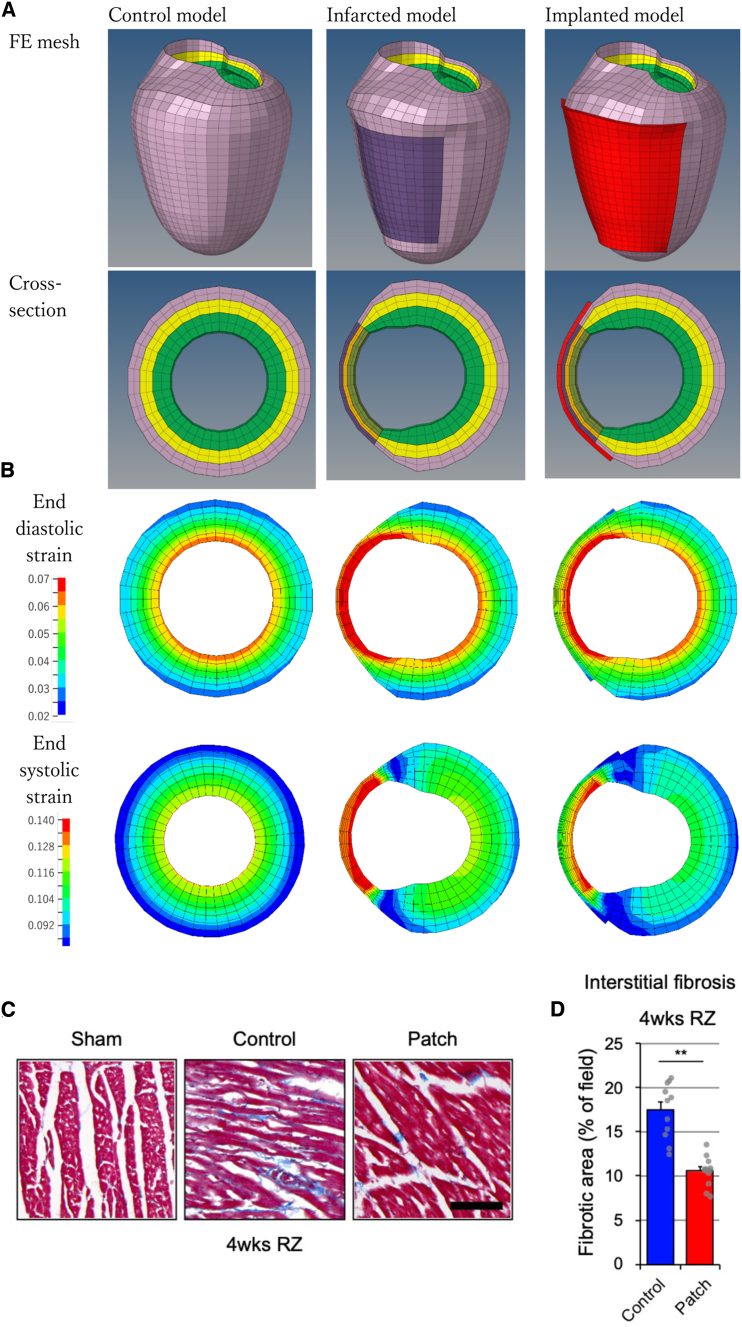
hiPSC-derived CM patch transplantation improved tissue elasticity (A and B) Finite element mesh and first primary strain distribution at LV of control, infarcted, and implanted models. (C) Interstitial fibrosis in the RZ visualized with Masson’s trichrome staining. Representative images from sham control, control, and patch-transplanted rats are shown. Scale bar, 100 μm. (D) Quantification of the interstitial fibrosis is shown. Fibrotic area was measured from the RZ of the rat 4 weeks after transplantation. *N* = 10 control, *N* = 13 transplanted rats. Two-tailed unpaired *t* test, ∗∗*p* < 0.01. Data represent mean ± SEM.

To evaluate the effect of altered material properties of the IZ by patch implantation on heart function, finite element results of control, infarcted, and patch-implanted models were compared (Figure 4A). The ejection fraction of the control model was 36.7%, in contrast to reduced values of 26.9% and 27.1% of the infarcted and implanted models, respectively. As shown in the small difference in the ejection fraction, stiffening of the infarcted zone largely unaltered the cardiac output. However, fiber directional strain showed a large difference between the infarct and implanted models. As shown in Figure 4B, strain in the infarcted zone was reduced (14%) at end-diastole. At end-systole, the strain was larger than in diastole. The patch treatment reduced the fiber directional strain by 11%, 7%, and 0.2% at IZ, border zone (BZ), and RZ. The largest mechanical change in IZ, moderate change in BZ, and small change in RZ accord with the observed collagen remodeling. In the IZ, rapid and substantial transcriptional changes were observed, whereas more modest transcriptional responses were detected in the BZ. In contrast, the RZ exhibited minimal transcriptional changes, suggesting that the observed remodeling in this region may occur through gradual accumulation of structural changes rather than immediate transcriptional responses. Tissue strain is considered a major mechanical signal that regulates the remodeling of post-ischemic hearts.^22^^,^^23^ Consistent with previous simulation studies,^22^^,^^24^^,^^25^^,^^26^^,^^27^^,^^28^ the stiffer infarct area produced by patch treatment relieved the strain to avoid pathologic remodeling. Consistently, significant improvement of interstitial fibrosis in the RZ was confirmed with Masson’s trichrome staining in the hiPSC-derived CM patch-transplanted group (Figures 4C and 4D). Additionally, transcription of the genes involved in collagen fibril formation was enriched at the IZ in the hiPSC-derived CM patch-transplanted group (Figure S4A), whereas it was increased at the RZ in control (Figure S4B). These results suggest that hiPSC-derived CM patch transplantation improves the quality of the scar, affecting myocardial elasticity to recover heart function.

### TGF-β signaling is upregulated in the hiPSC-derived CM patch-transplanted heart

**Figure 5:**
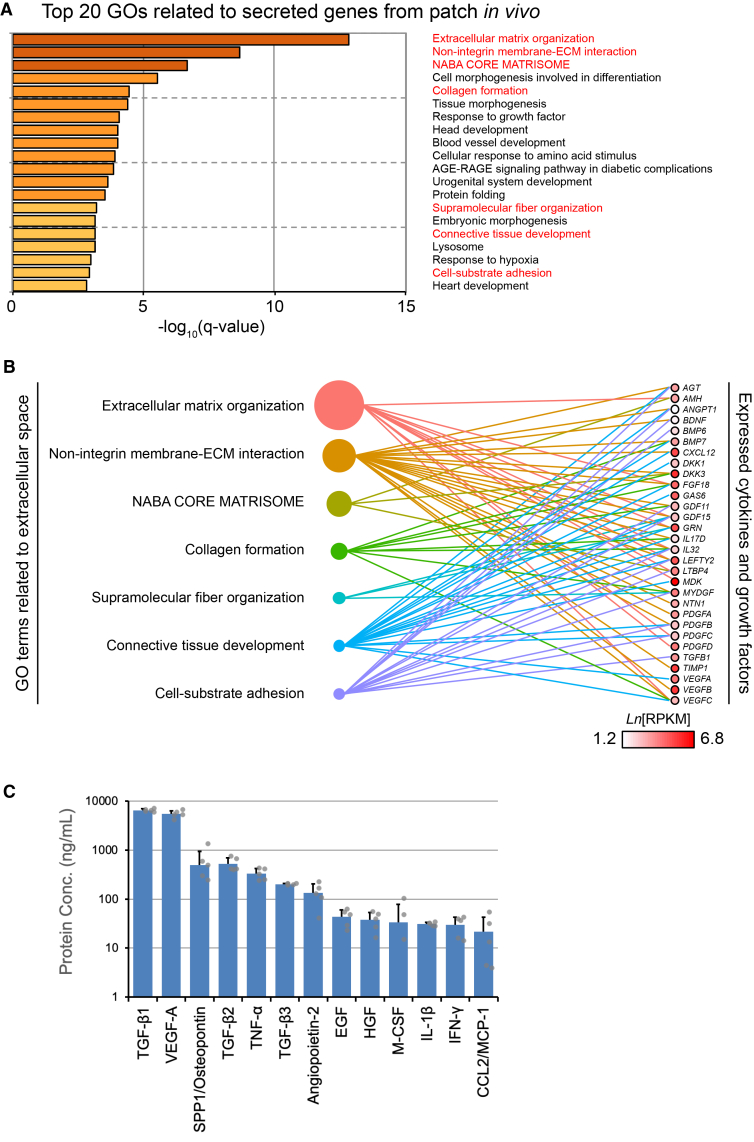
The hiPSC-derived CM patch secreted TGF-β *in vitro* and *in vivo* (A and B) Gene Ontology (GO) analysis of biological process. Metascape gene and functional annotation to produce gene cluster was examined. GO terms corresponding to biological process were extracted. Then, functional cluster analysis was carried out. Among the top 20 clusters, GOs focusing on secreted factors from hiPSC-derived CM patch are listed. (C) Secreted factors from hiPSC-derived CM patch *in vitro* were measured with ELISA. Top 13 factors identified are listed. Data represent mean ± SD. *N* = 5.

To gain mechanistic insight into hiPSC-derived CM patch transplantation, we examined transcriptome analysis with the cells isolated from the BZ of the rat heart. As the samples at 1 week post-transplantation showed significant enrichment of the genes involved in ECM remodeling, we examined RNA sequencing analysis with the samples at 1 week after surgery. Gene Ontology (GO) analysis focusing on 371 human secreted factors from the hiPSC-derived CM patch was carried out. Consistent with altered collagen expression in the transplanted heart, various GOs related to extracellular substrate synthesis and collagen biosynthesis correlated with each other (Figures 5A and 5B). To confirm *in vivo* observations, we measured the secreted factors from culture supernatant of the hiPSC-derived CM patch by the multiplex ELISA system. While various factors controlling angiogenesis, macrophage accumulation, or fibroblast activation were detected, TGF-β1, the major mediator of fibroblast activation,^29^^,^^30^ was identified with the highest score (Figure 5C).

**Figure 6:**
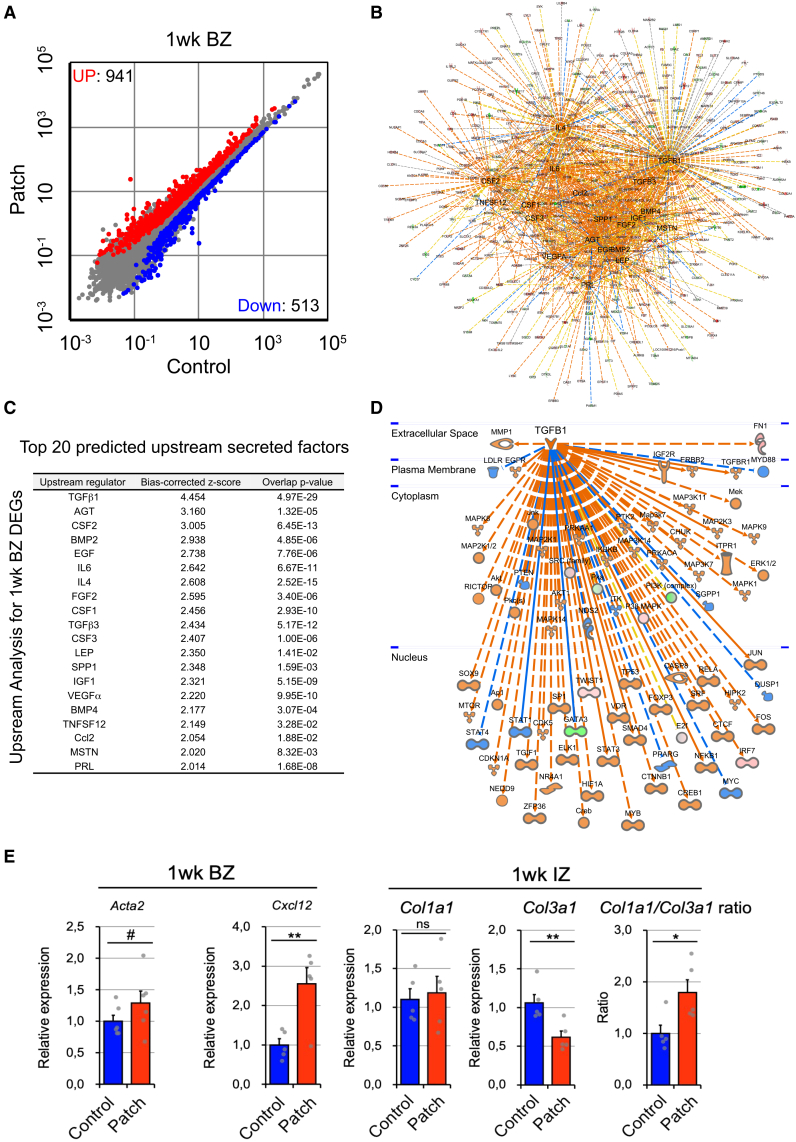
TGF-β signaling was upregulated in the host rat heart (A) Differentially expressed genes were enriched. Scatterplot showing the relationship between gene expression level with and without hiPSC-derived CM patch on the heart. The red dots and the blue dots represent the upregulated and downregulated genes, respectively. The gray dots represent the genes that were not differentially expressed. (B) Identified genes in (A) were analyzed by Ingenuity Pathway Analysis (IPA) upstream regulators. The summary network depicted the interactions between upstream regulators and downstream genes. (C) Based on the bias-corrected *Z* score and overlap *p* value, top 20 predicted upstream factors are shown with the scores. (D) The TGF-β1 pathway was identified with the highest score. All identified genes were overlaid onto the DEGs. (E) RT-qPCR analysis of *Acta2*, and *Cxcl12* in the sample derived from BZ, and *Cola1a1* and *Col3a1* in the sample derived from IZ in the transplanted heart. The ratio of *Cola1a1 and Col3a1* is also shown. Data represent mean ± SEM. *N* = 6 for the sample derived from BZ, and *N* = 5 for the sample derived from IZ. The signal was normalized with the value of *Actb*. Two-tailed unpaired *t* test, #*p* < 0.1, ∗*p* < 0.05, ∗∗*p* < 0.01.

Next, we analyzed gene expression profiles in the rat heart tissue. Meta-analysis was performed in comparison between 3 control rat hearts and 3 hiPSC-derived CM patch-transplanted hearts, and 1,454 differentially expressed genes (DEGs) were identified from 32,623 genes. Expression of 941 genes was significantly increased more than 1.5 times and that of 513 genes was significantly decreased more than 1.5 times (Figure 6A). Then, Ingenuity upstream regulator analysis in Ingenuity Pathway Analysis (IPA) was applied to the DEGs to identify the cascade of upstream transcriptional regulators. An overlap *p* value reflects potential upstream regulators based on a significant overlap between dataset genes and known targets regulated by potential transcriptional regulators. The activation *Z* score is used to predict upstream regulators based on a significant pattern match of up-/downregulation. While various pathways were highlighted (Figure 6B), TGF-β1 was identified with the highest score (Figures 6C and 6D).

To confirm the result of sequencing analysis, we next examined qPCR analysis using the hiPSC-derived CM patch-transplanted heart tissue. RNA samples were collected from the BZ of rat hearts transplanted with hiPSC-CMs. These samples were then analyzed using rat-specific TaqMan probes targeting *Acta2* and *CxCl12*, the genes encoding α-SMA and SDF-1, respectively, both of which are known factors downstream of TGF-β signaling. Consistent with the results of *in vitro* experiments and RNA sequencing analysis, expression levels of α-SMA and SDF-1 were elevated (Figure 6E). In addition, RNA samples were also collected from the IZ of the transplanted heart and analyzed by qPCR. The ratio of *Col1a1/Col3a1* type was found to be increased in hiPSC-CM-transplanted rat hearts.

### The hiPSC-derived CMs can directly activate cardiac fibroblasts inducing Col1a1 but not Col3 production

**Figure 7:**
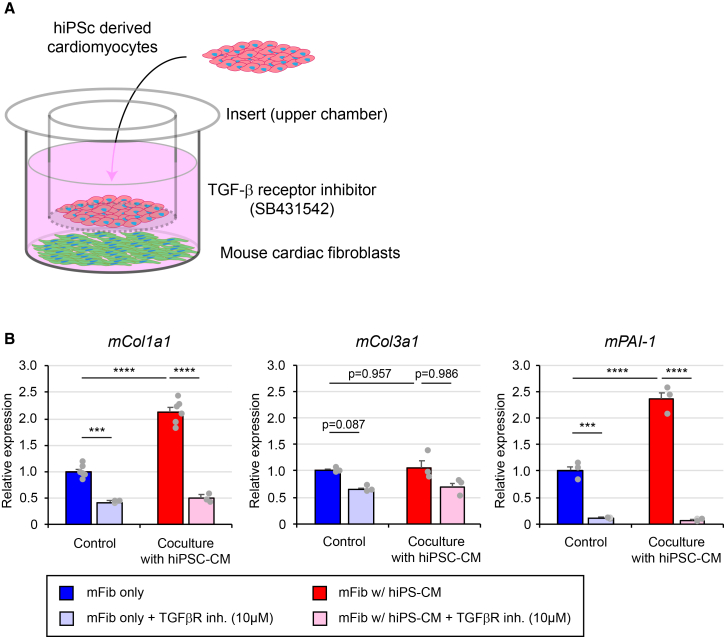
TGF-β secreted from the hiPSC-derived CM patch induced *Col1a1* expression but not *Col3a1* (A) Schematic representation of the co-culture system. hiPSC-derived CMs were seeded on the upper chamber, whereas the cardiac fibroblasts isolated from mice heart were cultured at the lower chamber in the presence and absence of SB431542, a TGF-β receptor inhibitor. (B) RT-qPCR analysis of *Col1a1*, *Col3a1*, and *Pai1* in the cultured cardiac fibroblasts. Two-way ANOVA (co-culture × inhibitor) with interaction; Tukey-adjusted post hoc tests on estimated marginal means. Data represent mean ± SEM. *N* = 3–6, one-way ANOVA followed by Tukey’s HSD test, *p* < 0.01, ∗∗∗*p* < 0.001, ∗∗∗∗*p* < 0.0001.

To further investigate the role of the hiPSC-derived CMs on collagen production, we established the co-culture system of hiPSC-derived CMs with cardiac fibroblasts. hiPSC-derived CMs were seeded onto the upper chamber of transwell, whereas cardiac fibroblasts isolated from adult mice were cultured at the lower chamber (Figure 7A). Plasminogen activator inhibitor type 1 (PAI-1) is an established downstream target of TGF-β across many cell types.^31^ When cardiac fibroblasts were co-cultured with hiPSC-derived CMs, *PAI-1*, a gene encoding PAI-1 protein, expression in the cardiac fibroblasts was significantly increased. In the presence of the TGF-β inhibitor, SB431542, *PAI-1* expression was strongly suppressed (Figure 7B). Two-way ANOVA revealed significant main effects and interactions for *Col1a1* and *PAI-1*, indicating that co-culture increased gene expression and this effect was effectively attenuated by SB431542. *Col3a1* showed similar trends but did not reach statistical significance. Under the condition, collagen production by cardiac fibroblasts was examined by quantitative reverse-transcription PCR (RT-qPCR). Co-culture of the cardiac fibroblasts with the hiPSC-derived CMs induced *Col1a1*, a gene encoding COL1A1, expression significantly. The treatment of the cells with SB431542 suppressed *Col1a1* expression in the presence and absence of the hiPSC-derived CMs (Figure 7B). Conversely, *Col3a1*, a gene encoding COL3A1, expression in the cardiac fibroblasts was not affected by co-culture with the hiPSC-derived CMs, although the basal level of *Col3a1* expression was decreased by treatment of the cells with SB431542 (Figure 7B). Of note, TGF-β expression in the cardiac fibroblasts was not affected by co-culture of cardiac fibroblasts with hiPSC-derived CMs (Figure S5). These results indicated that the hiPSC-derived CMs directly induced *Col1a1* expression but not *Col3a1* expression in the cardiac fibroblasts.

## Discussion

Stem cell therapies for heart failure are assumed as one of the potential treatment strategies after MI. Although improved parameters of cardiac performance were demonstrated after the transplantation of the cells, potential mechanisms underlying their beneficial effect still may be open for argument. In addition to stem cell potential to differentiate into CMs *in vivo*, paracrine effects of transplanted cells are demonstrated.^8^ However, a recent report suggests that the efficacy of stem cell transplantation, which is supposed to differentiate into CMs *in vivo*, is due to the tissue repairing process followed by tissue inflammation.^12^ The stem cells or the inflammatory reagent injection causes remodeling of the extracellular matrix of the IZ, resulting in enhanced mechanical properties of the damaged heart.^12^ On the other hand, the transplantation of exogenous CMs into the damaged heart is expected to increase contractility, thereby improving the heart function.^16^^,^^32^^,^^33^ While CMs derived from stem cells, such as hiPSCs, are immature in the cultured condition,^34^ grafted cells are thought to develop sarcomere and further differentiate after transplantation. Consistently, we observed maturation of CMs in the patch after transplantation (Figures 1D and 1E), which survived for at least 3 months. The sarcomeres of the transplanted cells at 12 weeks after transplantation were more mature than those at 4 weeks, whereas improved cardiac function was observed even at 4 weeks after transplantation. Sarcomere development and wall thickness may directly affect contractility of the heart. Interestingly, wall thickness of the IZ after 12 weeks did not show the correlation with functional recovery of the heart, although that after 4 weeks did. These results may suggest that wall thickness and CM maturation would not be critical for the functional recovery of the damaged heart after hiPSC-derived CM patch transplantation. On the other hand, we varied the patch-treated IZ material properties to assess the mechanical contribution of the patch itself. When the material properties were reduced to ½ or 1/5 of the original value, the averaged strain in the RZ decreased from 11% to 6.1% or 4.3%. As an extreme case, the model with patch-treated IZ properties but without the patch showed a 4.1% reduction in the IZ strain. If the stiffness of the patch is close to that of the IZ, as we assumed in the original material property, the patch contributes up to 60% of the reduction in the strain. The contribution of the patch decays fast with lesser stiffness.

Collagen fiber orientation and contents in the myocardial infarct scar have been shown to influence LV function.^35^ Here, we have found that hiPSC-derived CM patch transplantation induced production of collagen type I but not collagen type III via fibroblast activation through TGF-β secretion. Additionally, collagen fibers in the scar of the patch-transplanted group showed a directional and aligned organization of collagen fibers. These findings further support the idea that hiPSC-derived CM patch transplantation improves the mechanical property of the scar. As contractile force of the heart is generated by orchestrated movement of each CM, impaired stiffness of the IZ mechanically affects other parts of the heart including the RZ.^36^ Accordingly, fibrosis in the RZ increased after MI, which was improved in the patch-transplanted group (Figures 4C and 4D).

A previous report has shown that hiPSC-derived CM patch transplantation results in a better outcome of the heart function after MI compared to that of MSC transplantation.^37^ Fibrosis is a complicated process, in which many cell types are involved. Consistently, we have identified various secretion factors from the transplanted CM patch, which are responsible for changes such as triggering angiogenesis and blood cell migration. Given the direct effect of the hiPSC-derived CM patch on the cardiac fibroblast, our result may explain the mechanistic advantage of hiPSC-derived CM transplantation compared with other cell therapies for MI. Furthermore, our results suggest that the induction and modulation of fibrosis, previously considered a contributor to disease progression, may in fact play a role in the therapeutic mechanism. While fibrosis is generally regarded as detrimental in many organs, our findings indicate that controlled fibrosis may support tissue functional recovery. This insight opens the possibility that similar therapeutic strategies could be applied to fibrotic diseases in other organs.

### Limitations of the study

The study is constrained by some limitations. Because it is technically difficult to monitor how scar status affects the overall mechanical properties of the heart in living animals, we demonstrated this using computer simulations. Further research is required to address these issues. All experimental animals were male rats, and the hiPSC-derived CM patches were generated from a male donor. Therefore, potential sex-related differences or host-graft sex interactions could not be assessed. Future studies incorporating both sexes will be needed to address this point.

## Resource availability

### Lead contact

Requests for further information and resources should be directed to and will be fulfilled by the lead contact, Masanori Nakayama (masanori.nakayama@okayama-u.ac.jp).

### Materials availability

This study did not generate new unique reagents.

### Data and code availability

• RNA-seq data have been deposited in the Gene Expression Omnibus (GEO) and are publicly available as of the date of publication under accession number GSE318811. Accession number is also listed in the key resources table.
• This article does not report original code.
• Any additional information required to reanalyze the data reported in this paper is available from the lead contact upon request.

## Acknowledgments

Funding for this project was provided by the Excellence Cluster Cardio-Pulmonary System, the 10.13039/501100001659German Research Foundation, 10.13039/501100001659Deutsche Forschungsgemeinschaft, the 10.13039/100005156Alexander von Humboldt Foundation, Germany and Grants-in-Aid for Scientific Research Fund for the promotion of Joint International Research (Fostering Joint International Research (B)), JSPS, Japan.

## Author contributions

K.T., R.M., U.K., and M.N. conceptualized and designed the study. K.T., R.M., and H.T. performed the majority of the experimental work. K.T. performed bioinformatic analyses. F.N., H.S., S.I., and A.H. performed mechanical simulations of cardiac tissue models. M.T., T.H., and H.I. prepared histological samples and analyzed the data. K.T. and R.M. wrote the original draft of the manuscript. K.T., R.M., T.H., T.B., Y.S., S.M., and M.N. reviewed the data and edited the manuscript. All authors have read and agreed on the published version of the manuscript.

## Declaration of interests

Y.S. is a founder and chief technology officer of Cuorips Inc., a company developing cardiac regenerative therapies related to the technology described in this study. Some authors are affiliated with Cuorips Inc. or have financial interests in the company. K.T. and U.K. were previously employed by Daiichi Sankyo Co., Ltd. and were seconded to Cuorips Inc. during the period when this study was conducted but currently have no employment relationship with and hold no equity in Cuorips Inc. No other competing interests are declared.

## STAR★Methods

### Key resources table

**Table:** 

REAGENT or RESOURCE	SOURCE	IDENTIFIER
**Antibodies**		

Human nuclei antigen	Merk Millipore	Cat# MAB1281; RRID: AB_94090
MLC2a	Synaptic Systems	Cat# 311 011; RRID: AB_887737
MLC2v	Synaptic Systems	Cat# 310 111; RRID: AB_887738
Sarcomeric Alpha Actinin	Abcam	Cat# ab68167; RRID: AB_11157538
Cardiac troponin-T	Abcam	Cat# ab45932; RRID: AB_956386
Col1a1	OriGene	Cat# R1038; RRID: AB_522921
Col3a1	Abcam	Cat# ab6310; RRID: AB_305413
GADPH	Thermo Fisher Scientific	Cat# AM4300; RRID: AB_2536381
Anti-mouse IgG Alexa Fluor 488	Thermo Fisher Scientific	Cat# A11029; RRID: AB_2534088
Anti-rabbit IgG Alexa Fluor 568	Thermo Fisher Scientific	Cat# A11036; RRID: AB_10563566

**Chemicals, peptides, and recombinant proteins**		

CHIR99021	LC Laboratories	Cat# C-6556
Wnt-C59	Selleck Chemicals	Cat# S7037
XAV939	Sigma-Aldrich	Cat# X3004
L-lactic acid	FUJIFILM Wako Pure Chemical Corporation	Cat# 129-02666
L-glutamine	Thermo Fisher Scientific	Cat# 25030081
Primate embryonic stem cell media	ReproCELL	Cat# RCHEMD001
StemPro-SFM34	Thermo Fisher Scientific	Cat# 10639011
1-thioglycerol	Sigma-Aldrich	Cat# M6145
Accumax	Nacalai Tesque	Cat# 17087-54
L(+)-ascorbic acid	FUJIFILM Wako Pure Chemical Corporation	Cat# 014-04801
2,4,6-Trinitrophenol	FUJIFILM Wako Pure Chemical Corporation	Cat# 209-08675
Sirius red	PolySciences	Cat# 09400-10
T-PER tissue protein extraction reagent	Thermo Fisher Scientific	Cat# 78510
Protease Inhibitor Cocktail	Nacalai Tesque	Cat# 04080-11
NuPAGE LDS Sample Buffer (4X)	Thermo Fisher Scientific	Cat# NP0007
Collagenase type 2	Worthington Biochemicals Corporation	Cat# LS004176
Trypsin (2.5%), no phenol red	Thermo Fisher Scientific	Cat# 15090-046
HBSS	Sigma-Aldrich	Cat# H6648
FGM-3 Cardiac Fibroblast Growth Medium-3 BulletKit	Lonza	Cat# CC-4526
Gelatin Solution	Sigma-Aldrich	Cat# G1393
SB 431542	Bio-Techne	Cat# 1614
MitoTracker Red CMXRos	Thermo Fisher Scientific	Cat# M7512
RPMI 1640 Medium, no glucose	Thermo Fisher Scientific	Cat# 11879020
DMEM, no glucose	Thermo Fisher Scientific	Cat# 11966-025
DMEM	Nacalai Tesque	Cat# 08458-45
Fetal Bovine Serum	Thermo Fisher Scientific	Cat# 26140-079
StemFit AK02N	Ajinomoto Healthy Supply	Cat# RCAK02N
iMatrix-511	Matrixome	Cat# 892012
TrypLE Select Enzyme (1X), no phenol red	Thermo Fisher Scientific	Cat# 12563011
DMEM/F-12, GlutaMAX supplement	Thermo Fisher Scientific	Cat# 10565018
Penicillin-Streptomycin	Thermo Fisher Scientific	Cat# 15140122
BMP4	Bio-Techne	Cat# AFL314E
Activin A	Bio-Techne	Cat# AFL338
FGF basic	Bio-Techne	Cat# AFL233
FGF basic	ReproCELL	Cat# RCHEOT002
VEGF	Bio-Techne	Cat# BT-VEGF-AFL
IWR-1	Sigma-Aldrich	Cat# I0161
IWP-2	Sigma-Aldrich	Cat# I0536
Y-27632	FUJIFILM Wako Pure Chemical Corporation	Cat# 259-00613
Potassium Chloride	Nacalai Tesque	Cat# 28514-75
4%-Paraformaldehyde Phosphate Buffer Solution	Nacalai Tesque	Cat# 09154-85
VECTASHIELD Mounting Medium with DAPI	Vector Laboratories	Cat# H-1200
OCT compound	Sakura Finetek Japan	Cat# 45833
Sucrose	Nacalai Tesque	Cat# 30406-25
PBS, pH 7.4	Thermo Fisher Scientific	Cat# 10010023

**Critical commercial assays**		

RNeasy Mini Kit	Qiagen	Cat# 74106
TruSeq stranded mRNA sample prep kit	Illuminia	Cat# RS-122-2101
Quick-RNA Miniprep Kit	Zymo Research	Cat# R1055
SuperScript VILO cDNA synthesis kit	Thermo Fisher Scientific	Cat# 11754050
PowerUp SYBR Green Master Mix	Thermo Fisher Scientific	Cat# A25742
Bio-Plex Pro human cytokine group-I 27-Plex panel	Bio-Rad Laboratories	Cat# M500KCAF0Y
Bio-Plex Pro Human cytokine group-II 21-Plex panel	Bio-Rad Laboratories	Cat# MF0005KMII
Bio-Plex Pro TGF-β 3-Plex panel	Bio-Rad Laboratories	Cat# 171-W4001M
Bio-Plex Pro Human cancer biomarker 1 16-Plex panel	Bio-Rad Laboratories	Cat# 171-AC500M
Bio-Plex Pro Human cancer biomarker 2 18-Plex panel	Bio-Rad Laboratories	Cat# 171-AC600M

**Deposited data**		

Processed bulk RNA-seq data	GEO (This paper)	GSE318811

**Experimental models: Cell lines**		

Ff-I14 Human iPSCs	Kyoto University	RRID:CVCL_C1DM
253G1 Human iPSCs	Kyoto University	RRID:CVCL_B518

**Experimental models: Organisms/strains**		

Immunodeficient rat (nude rat)	CLEA Japan	F344/NJcl-rnu/rnu
C57BL/6	CLEA Japan	N/A

**Oligonucleotides**		

mCol1a1 forward primer 5′- GCTCCTCTTAGGGGCCACT-3′ and reverse primer 5′- CCACGTCTCACCATTGGGG-3′	This paper	N/A
mCol3a1 forward primer 5′- CTGTAACATGGAAACTGGGGAAA-3′ and reverse primer 5′- CCATAGCTGAACTGAAAACCACC-3′	This paper	N/A
mPai1 forward primer 5′- TTCAGCCCTTGCTTGCCTC-3′ and reverse primer 5′- ACACTTTTACTCCGAAGTCGGT-3′	This paper	N/A
mTgfb1 forward primer 5′- TGGAGCAACATGTGGAACTC-3′ and reverse primer 5′- GTCAGCAGCCGGTTACCA-3′	This paper	N/A
mHprt forward primer 5′- TCCTCCTCAGACCGCTTTT-3′ and reverse primer 5′- AACCTGGTTCATCATCGCTAA-3′	This paper	N/A

**Software and algorithms**		

Excel 2010 ver.14.0.	Microsoft Corporation	N/A
Illumina Casava ver.1.8.2 software	Illumina	RRID:SCR_001802
FastQC ver.0.7.2	Babraham Institute	RRID:SCR_014583
Trim Galore ver.0.5.0	Babraham Institute	RRID:SCR_011847
HISAT2 ver.2.1.0	Johns Hopkins University	RRID:SCR_015530
Subread ver.1.6.2	University of Melbourne	RRID:SCR_009803
Subio platform ver.1.22	Subio	N/A
DESeq2 package ver.1.26.0 of R software 3.6.2	R foundation	RRID:SCR_015687
GSEA software ver.3.0	Broad Institute	RRID:SCR_003199
Ingenuity Pathway Analysis software	Qiagen	RRID:SCR_008653
Metascape ver.3.5	Metascape.org	RRID:SCR_016620
ImageJ ver1.52a	National Institutes of Health	RRID:SCR_003070
JMP ver. 10.0.0	SAS Institute	RRID:SCR_014242

**Other**		

Echocardiography	Hitachi-Aloka medical	SSD-F75
15-MHz phased-array transducer	Hitachi-Aloka medical	UST-5417
Micromanometer-tipped catheter	Millar Instruments	SPC-320
Desktop microscope	Keyence	BIOREVO BZ-9000
Fluorescence microscope	Thermo Fisher Scientific	EVOS M-700
Disrupter	TOMY	MS-100R
RNA quantification/quality control	Agilent Technologies	Bioanalyzer 2100
ELISA	Bio-Rad Laboratories	Bio-Plex 200
Cryostat	Leica Microsystems	CM3050S
Microscope	Olympus	BZ50 microscope
Homogenizer	Nippi	320103
Chemiluminescence imager	Amersham	Imager 600
Microscope	Carl Zeiss	Axio Scan.Z1 Digital Slide Scanner

### Experimental model and study participant details

#### Rat models

Six-to eight-week-old male nude rats (F344/NJcl-rnu/rnu; CLEA Japan, Tokyo, Japan) were used in this study. All animal experiments were conducted in accordance with institutional guidelines and were approved by the animal ethics committee of ASUBIO PHARMA Co., Ltd. (approval number: HG-14-001). These rats were maintained in a specific pathogen–free room in our animal facility on a 12-h light/12-dark cycle with free access to food and water. Only male rats were used in this study. Therefore, potential sex-based differences were not evaluated.

#### Human induced pluripotent stem cells

Human iPSC-derived cardiomyocyte patches were generated from male donor–derived human iPSC lines (Ff-I14 and 253G1) obtained from the Center for iPS Cell Research and Application (CiRA), Kyoto University, Japan. The iPSC lines were previously established and no new human participants were recruited for this study. The identity of the human iPSC lines was authenticated by the provider (CiRA). All cell lines were routinely tested for mycoplasma contamination and were confirmed to be negative. Because only male donor–derived iPSC lines were used, potential sex-based differences were not evaluated in this study.

### Method details

#### Preparation of hiPSC-derived CM patches

The hiPSC-derived CMs were first induced by using the previously reported chemically defined differentiation methods,^38^ then applied with the lactate method to purify cardiomyocytes.^39^ Briefly, Ff-I14 human iPSCs were cultured and maintained in StemFit AK02N medium (RCAK02NAK, Ajinomoto Healthy Supply, Tokyo, Japan) on iMatrix-511 (892012, Matrixome, Osaka, Japan), then were treated by CDM3 media and were treated by CDM3 media supplemented with 6 μM CHIR99021 (C-6556, LC Laboratories, Woburn, MA) for 2 days (day 0–2), then 2 μM Wnt-C59 (S7037, Selleck Chemicals, Houston, TX) and 3 μM XAV939 (X3004, Sigma-Aldrich, St. Louis, MO) for 2 days (day 3–4) and maintained for another 11 days (day 5–16). The differentiated cells were cultured to Dulbecco’s Modified Eagle Medium (DMEM) (11966-025, Thermo Fisher Scientific, Waltham, MA) supplemented with 4 mM L-lactate acid (129–02666,FUJIFILM Wako Pure Chemical Corporation, Osaka, Japan) instead of glucose as the carbon source for 3 days (day 16–18) to eliminate undifferentiated cells. Obtained CMs were plated in thermo-responsive culture 35 mm dishes (CS3007, CellSeed, Tokyo, Japan) at 4.0 × 10^6^ cells/dish density. CMs were cultured for 4 days in DMEM(08458-45, Nacalai Tesque, Kyoto, Japan), supplemented with 10% fetal bovine serum. The culture medium was changed twice (day 1 and 3) during patch formation.

#### Cardiac differentiation of human induced pluripotent stem cells (hiPSCs)

hiPSCs (253G1; Riken, Ibaraki, Japan) were used in this study. Undifferentiated hiPSCs were cultured and maintained in primate embryonic stem (ES) cell media (RCHEMD001, ReproCELL, Kanagawa, Japan) with basic fibroblast growth factor (bFGF) RCHEMD001, ReproCELL, Kanagawa, Japan) on mouse embryonic fibroblast cells (RCHEMD001, ReproCELL, Kanagawa, Japan). Cardiomyogenic differentiation was induced as previously described^40^ with some modifications. Cardiac differentiation was induced in StemPro-SFM34 (25030081, Thermo Fisher Scientific, Waltham, MA, USA) containing 2 mM L-glutamine (25030081, Thermo Fisher Scientific, Waltham, MA, USA)), 50 μg/mL ascorbic acid (014–04801, FUJIFILM Wako Pure Chemical Corporation), and 400 μM 1-thioglycerol (M6145, Sigma-Aldrich, St. Louis, MO, USA). hiPSCs were dissociated using Accumax ((17087-54, Tesque, Kyoto, Japan) and transferred to a bioreactor (ABLE Corporation &Biott Co., Japan) and were supplemented with several human recombinant proteins, including bone morphologic protein 4 (BMP4) (AFL314E, Bio-Techne, Minneapolis, MN, USA), activin A (AFL338, Bio-Techne, Minneapolis, MN, USA), bFGF (AFL233, Bio-Techne, Minneapolis, MN, USA), and vascular endothelial growth factor (VEGF) ((BT-VEGF-AFL, Bio-Thecne, Minneapolis, MN, USA), and small molecules such as IWR-1 (I0161, Sigma-Aldrich, St. Louis, MO, USA) and IWP-2 (I0536, Sigma-Aldrich, St. Louis, MO, USA)). Each protein was used at the corresponding days: days 0–1, BMP4; days1–4, activin A, BMP4 and bFGF; days 4–6, IWR-1 and IWP-2; after day 6, VEGF and bFGF.

#### Cell patch transplantation after MI with immunodeficient rats

Six-to eight-week-old male nude rats (F344/NJcl-rnu/rnu; CLEA Japan, Tokyo, Japan) were used in the experiments as described previously.^41^^,^^42^ Briefly, these rats were purchased from CLEA Japan, Inc (Tokyo, Japan). All animal experiments were conducted according to protocols approved by the animal ethics committee of ASUBIO PHARMA Co., Ltd. (approval number: HG-14-001). The rats were anesthetized with endotracheal intubation using isoflurane (2%). A left thoracotomy was performed, and the left anterior descending artery was permanently ligated with a 5-0 nylon monofilament. Successful ligation was confirmed by the color change of the anterior left ventricular (LV) wall. After 1 week, rats underwent re-thoracotomy for the exposed pericardial space and were then randomly assigned into a control (without patch transplantation) group or a patch transplanted group. hiPSC-derived CM patches were transplanted over the epicardium of the anterior and lateral left-ventricular walls. hiPSC-derived CM patches were spread manually to cover both infarct and border areas of the heart and fixed by covering with pericardial membrane. The chest was closed with a 4-0 nylon monofilament. After surgery, all rats were allowed to recover in individual temperature-controlled cages. These rats were maintained in a specific pathogen–free room in our animal facility on a 12-h light/12-dark cycle with free access to food and water. Rats were euthanized each day after surgery, and the heart specimens were harvested.

#### Functional analysis of the rat heart

##### Echocardiography

Echocardiography (SSD-F75, Hitachi-Aloka medical, Tokyo, Japan) was performed under anesthesia with isoflurane (2%). Images were recorded using a 15-MHz phased-array transducer (UST-5417, Hitachi-Aloka medical, Tokyo, Japan). End-diastolic and end-systolic diameters of the left ventricle (EDD and ESD, respectively) were derived from M-mode tracings, obtained from parasternal short-axis views.

##### Catheterization

The right carotid artery was cannulated with a micromanometer-tipped catheter (SPC-320, Millar Instruments, Houston, TX) that advanced into the left ventricle via the aorta for recording left ventricular pressure and dP/dt. Isoflurane (2%) was used for anesthesia.

##### Passive pressure-volume analysis

*In vitro* LV pressure–volume curve was measured as described previously,^43^ with some modifications. The heart was arrested by an injection of KCl and then quickly excised. A double-lumen catheter was inserted into the left ventricle, which was isolated by ligating the atrioventricular groove. Reproducible pressure–volume curves were generated over a pressure range of 0–30 mmHg by infusing saline at a speed of 0.4 mL/min.

All the outcomes were analyzed by unpaired *t* test analysis to compare groups at each time point. Continuous data are presented as mean ± SEM. Statistical comparisons were performed by unpaired *t* test. *p* value <0.05 were considered statistically significant. *p*-values were adjusted for multiple testing using the Bonferroni correction. All analyses were performed using Excel 2010 ver. 14.0. (Microsoft Corporation. Redmond WA).

##### Immunohistolabeling and fluorescence-intensity analysis

Harvested hearts were fixed with 4% paraformaldehyde (09154-85, Nacalai Tesque, Kyoto, Japan), then processed to prepare frozen sections (see histological analysis section for details). Each frozen section was stained with primary antibodies, followed by incubation with fluorescence-conjugated secondary antibodies, and counterstaining with 4′, 6-diamidino-2- phenylindole (DAPI) (H-1200, Vector Laboratories, Newark, CA, USA). Finally, fluorescence microscopy images were captured by BIOREVO BZ-9000 (Keyence, Osaka, Japan) or EVOS M-700 (Thermo Fischer Scientific, Waltham, MA). Fluorescence dye and antibodies were used as follows; MitoTracker Red CMXRos (for prestaining of patch before surgery) (M7512, Thermo Fisher Scientific, Waltham, MA), human nuclei antigen (MAB1281, Merk Millipore, Burlington, MA), MLC2a (311 011, Synaptic Systems, Göttingen, Germany), MLC2v (310 111, Synaptic Systems, Göttingen, Germany) sarcomeric actinin (ab68167, Abcam, Cambridge, UK), and cardiac troponin-T (mainly reacts with human protein) (ab45932, Abcam, Cambridge, UK). Fluorescence-conjugated secondary antibodies were used as follows; goat anti-mouse IgG Alexa Fluor 488(A11029, Thermo Fischer Scientific, Waltham, MA) and goat anti-rabbit IgG Alexa Fluor 568 (A11036, Thermo Fischer Scientific, Waltham, MA).

##### Finite element model of normal, infarct, and hiPSC-patch implanted left ventricle

We used simplified LV model geometry as in the previous study.^24^ Since fiber orientation of LV varies across the wall, the ventricular wall was divided into three layers and fiber orientations were set at −60, 0, and 60° respectively. Local coordinates were defined by element edge-based with x as radial edge direction, y as in-surface-plane direction, and z perpendicular to the x-y plane. Fiber direction was defined in the x-y plane with x-direction as 0° and y-direction as 90°. For the infarction model, 13.4% of the ventricular wall was thinned to 1/3 of the original thickness to represent the morphological change. The hiPSC patch was modeled to cover 22.8% of the ventricular wall.

#### Material model

To characterize the material properties of cardiac muscle tissue, we adopted a transversely isotropic model by Walker et al.^44^ for the constitutive equation. The stress *σ* is calculated as the sum of the passive stress *σ*_pass_ and the active stress *T*; *σ* = *σ*_pass_+*T*. Passive stress is based on hyperelastic material theory, in which the stress-strain relationship derives from a strain energy potential (W) as in Equation 1.

(Equation 1) $${\sigma \;}_{\text{pass}}=\frac{1}{J}FSF^{T}=\frac{1}{J}F\left(\frac{\partial W}{\partial E}-pJC^{-1}\right)F^{T}\text{,}$$

where sigma is Cauchy stress, *S* is second Piola-Kirchoff stress, *F* is deformation gradient tensor, *E* is Green -Lagrange strain, *C* is right Cauchy-Green deformation tensor, *p* is static pressure, and *J* = det(*F*). W is defined as follows:

(Equation 2) $$W=\frac{C}{2}\left(e^{Q}-1\right)$$

(Equation 3) $$Q=b_{1}E_{11}^{2}+b_{2}\left(E_{22}^{2}+E_{33}^{2}+E_{23}^{2}+E_{32}^{2}\right)+b_{3}\left(E_{12}^{2}+E_{21}^{2}+E_{13}^{2}+E_{31}^{2}\right)$$

where *C*, *b*_1_, *b*_2_, and *b*_3_ were material parameters. For healthy cardiac muscle tissue, the material parameter values were adopted from Walker et al.^44^ For the infarct region and patch-implanted infarcted region, *b*_1_, *b*_2_, and *b*_3_ were fitted to reproduce the decrease of force development to 35% and 49% at 0.2 strain compared to that of the healthy cardiac tissue reported by Vagnozzi et al.^12^ For the patch itself, we assumed that the fiber structure is immature and the material is isotropic within a patch plane. The parameters were fitted under the assumption that the uniaxial force-length relationship of the patch is equivalent to that of the infarct region. The material parameters were shown in the table and the uniaxial force-length relationships of the healthy, infarct, implanted and patch models were shown in Figure S1.

Active stress *T* is defined with fiber directional component *T*_0_ and transverse components *T*_1_ = 0.4*T*_0_. *T*_0_ is defined as length-dependent as follows:

$$T_{0}=T_{\max}\frac{Ca_{0}^{2}}{Ca_{0}^{2}+ECa_{50}^{2}}\cdot \frac{1}{2}\left(1-\cos\;w\right)\text{,}$$

$$ECa_{50}=\frac{{\left(Ca_{0}\right)}_{\max}}{\sqrt{\exp\left[B\left(l-l_{0}\right)\right]-1}},l=l_{R}\sqrt{2E_{11}+1},w=\pi \frac{0.25+t_{r}}{t_{r}},t_{r}=ml+b$$

where *T*_*max*_ is the maximum isometric tension achieved at the longest sarcomere length, ${\left(Ca_{0}\right)}_{\max}$ is maximum peak intracellular calcium concentration, *l*_0_ is the sarcomere length at which no active tension develops, *l*_*R*_ is the stress-free sarcomere length, and *B*, *m*, and *b* are constants. Material parameters were set following the previous study by Walker et al.^44^ and shown in the following table.

**Table:** Constitutive model parameters

	healthy	infarcted	implanted	patch
density $\left[\text{kg}/m^{3}\right]$	$1.37\times 10^{3}$			
C[Pa]	359			
$b_{1}$[−]	67.07	46.80	53.40	50.00
$b_{2}$[−]	24.16	22.00	22.00	50.00
$b_{3}$[−]	21.60	18.00	18.00	21.60

**Table:** Material parameters for length-dependent active tension development

$T_{\max}$[kPa]	$1.357\times 10^{2}$
$l_{0}$[μm]	1.58
$l_{R}$[μm]	1.91
B[/μm]	4.75
m[s/μm]	1.049
bb[s]	−1.429
$Ca_{0}$[μmol/l]	4.35
${\left(Ca_{0}\right)}_{\max}$[μmol/l]	4.35

#### Simulation

We conducted two static simulations representing end-diastole and end-systole. At end-diastole, a diastolic pressure of Ped = 1500 Pa was applied to the endocardial surface without any active force generation in the cardiac tissue (T = 0). At end-systole, an end-systolic pressure (Pes) was applied together with active force generation in the ventricular tissue, assuming maximum intracellular calcium concentration. Therefore, we did not explore a range of pressures or active contraction forces, but rather simulated only these two discrete conditions.

To clarify the relationship between the material models and the simulation settings, we have revised the text as follows. For end-diastole: A diastolic pressure of Ped = 1500 Pa was applied to the endocardium without active force development (T = 0). For end-systole: An end-systolic pressure of Pes (13,000 Pa for the healthy model and 10,000 Pa for the infarct and implanted models) was applied, along with maximum active force development (T calculated with Ca_0_ = Ca_0(max)_).

#### RNA-sequence analysis

Each snap frozen heart tissue (timepoint; Day3, 1week, 4weeks, tissue; IZ, BZ, RZ for each group) were homogenized in Buffer RLT by disrupter MS-100R (TOMY, Tokyo, Japan). Total-RNA was extracted using RNeasy Mini Kit (74106, Qiagen, Venlo, Netherlands) according to the manufacturer’s instructions. The quality of purified RNAs was assessed by Bioanalyzer 2100 (Agilent Technologies, Santa Clara, CA), and then proceeded to downstream analyses.

Library preparation was performed using a TruSeq stranded mRNA sample prep kit (RS-122-2101, Illumina, San Diego, CA) according to the manufacturer’s instructions. Whole transcriptome sequencing was applied to the RNA samples on an Illumina HiSeq 2500 platform in a 75-base single-end mode. Illumina Casava ver.1.8.2 software was used for base calling. Obtained reads were quality filtered by FastQC ver. 0.7.2 and Trim Galore ver. 0.5.0, then aligned to in silico combined human (GRCh37)-rat (Rattus norvegicus, Rnor_6.0) by HISAT2 ver.2.1.0^45^ with default setting as described previously.^46^ The uniquely aligned reads were annotated to genes and calculated read-count by featureCounts function of Subread ver. 1.6.2^47^ for human and rat, respectively. Subio platform ver. 1.22 was used for visualizing RPKM (reads per kilobase of gene per million mapped reads) values of each sample. Differentially expressed genes (DEGs, fold-change >1.5, raw *p*-value <0.05) were extracted by DESeq2 package ver. 1.26.0^48^ of R software ver.3.6.2 (https://www.R-project.org/).

Gene set enrichment analysis (GSEA) was performed by GSEA software ver. 3.0^49^ using pre-ranked mode with default setting. The pre-ranked gene lists were generated by log2 fold-change value obtained from DEGs-calling described above. Pre-defined official gene-sets (e.g., Gene Ontology (GO) categories) were obtained from Molecular Signatures Database ver. 6.2 Website.

To predict upstream humoral factors which were consistent with host rat heart transcriptome changes upon hiPSC-derived CMs patch transplantation, Upstream regulator analysis function of Ingenuity Pathway Analysis (IPA) software (Qiagen, Venlo, Netherlands) was used to calculate consistence of gene regulatory relationships of knowledge-based statics. DEGs of 1week_BZ (patch vs. control) were imported to IPA software, then statistically predicted upstream regulators were extracted for molecular type “cytokine” and “growth factor”, bias-collected *Z* score <2.0, overlap *p*-value <0.05. Then the network of predicted humoral factors and their downstream genes were visualized.

To assess functional significance of genes secreted from hiPSC-derived CMs patch after transplantation, gene ontology analysis was carried out by using Metascape ver. 3.5^50^ with default setting. Possible secreted genes were extracted from all expressed human transcripts which were detected in RNA-seq experiment by “Secreted” tag of annotation from UniPlot and Protein Atlas. The “Secreted” 1,305 gene in all Ensembl genes were imported as background genes for gene ontology analysis.

#### Multiplex ELISA measurement

To evaluate humoral factors secreted by hiPSC-derived CM patch, conditioned culture media of hiPSC-derived CM patch was sampled during patch formation, then dozens of humoral factors in culture supernatant were measured on bead-based multiplexed ELISA system, Bio-Plex 200 (Bio-Rad Laboratories, Hercules, CA), according to manufacturer’s instructions. The tested assay panels were as follows; human cytokine group-I 27-Plex (M500KCAF0Y), human cytokine group-II 21-Plex (MF0005KMII), TGF-β 3-Plex panel (171-W4001M), human cancer biomarker 1 16-Plex (171-AC500M), and human cancer biomarker 2 18-Plex (171-AC600M). For background correction, the signals of non-treated medium were subtracted from each measurement.

#### Histological analysis

Harvested rat left ventricle was fixed with 4% paraformaldehyde at 4C° overnight and stored in PBS(−) (10010023, Thermo Fisher Scientific, Waltham, MA). The left ventricle was cut into four pieces in short-axis direction. The cut tissues were embedded and frozen in OTC compound (45833, Sakura Finetek Japan, Tokyo, Japan) on cryomold plates after infiltration with 30% sucrose/PBS(−) (10010023, Thermo Fisher Scientific, Waltham, MA) as a cryoprotectant before freezing with pulverized dry ice. The frozen blocks were sliced at 4 μm-thick on the cryostat CM3050S (Leica Microsystems, Wetzlar Germany). The frozen sections were used for immunofluorescence imaging (see described above), Masson’s Trichrome staining and Picro-Sirius Red staining. In brief, Masson’s trichrome staining was carried out as follow; Weigert’s iron hematoxylin staining was carried out to stain the nuclei in black, and Biebrich scarlet-acid fuchsin solution was used to stain cytoplasm and muscle fibers in red. After treatment with phosphotungstic acid, collagen is stained in blue with aniline blue. Picro-Sirius Red staining was carried out by treating with 2,4,6-Trinitrophenol (209–08675, FUJIFILM Wako Pure Chemical Corporation, Osaka, Japan) and sirius red stain (09400-10, PolyScience, Niles, IL) for 5–10 min.

#### Scar size measurement

To determine the infarct size of each animal, all sections from each heart were digitized by Axio Scan.Z1 Digital Slide Scanner (Carl Zeiss, Oberkochen, Germany). The ratio of the entire length of infarct area (both endo- and epicardium) of left ventricular free wall against the entire length of endo- and epicardium of left ventricular wall (including interventricular septum) of four sections was measured using Masson’s trichrome stained sections by ImageJ (ver 1.52a).

#### Wall thickness measurement

To determine the wall thickness of each animal, the ratio of average thickness of randomly selected 10 points of infarct area of left ventricular free wall (including only host rat myocardium) against that of interventricular septum of four sections were measured using digitized slices of Masson’s trichrome stained sections by ImageJ software (ver 1.52a). To evaluate the contribution of engrafted hiPSC-derived CM patch to wall thickness, we measured the thickness of infarct area of left ventricular free-wall with engrafted tissue if the transplanted tissues existed in randomly selected measuring points. The transplanted tissues were distinguished by immunofluorescence imaging of human nuclear antigen using serial section of Masson’s trichrome stained samples. For correlation analysis between wall thickness and heart function, wall thickness values calculated above were plotted against indices of heart function, then statistical correlation was tested for Pearson product-moment correlation coefficient by using JMP ver. 10.0.0 (SAS Institute Inc.).

#### Polarized imaging of fibrotic tissue

To evaluate physical maturation of scar tissue, we used linear polarized light microscopy of Picro-Sirius Red stained sections. The polarized images of the middle of infarct area of each section were captured using BZ50 microscope (Olympus, Tokyo, Japan) with polarized optics (5 images per animal). Then, the polarized images were binarized to separate “green-yellow” (mainly consists of immature collagen fibers) and “orange-red” (mainly consists of well-organized matured collagen fibers) area, respectively with pre-defined thresholds of Lab color space using ImageJ software (ver 1.52a). Finally, scar maturation was expressed as an average ratio of “orange-red” area against “green-yellow” area for each animal. Collagen fiber angle measurements were binned into 5-degree bins^35^ to produce histogram representing collagen fiber orientation. This data was then normalized by taking the median peak and shifting it to the 0-degree orientation, while simultaneously shifting all other values by the equivalent amount. Unpaired two-tailed *t* tests were performed to compare the percentage of fibers oriented in 0 ± 5° for all biological replicates.

#### Measurement of interstitial fibrosis

To evaluate interstitial fibrosis of heart tissue, we captured high magnitude images of interventricular septum using digitized slices of Masson’s trichrome stained sections (6 images per animal). To avoid bias of the collagen contents derived from elastic fibers, we selected images which did not contain mature vascular tubes. Then, the captured images were binarized to separate “blue” (mainly consists of fibrotic tissue) and whole tissue area including fibrous tissue, respectively with pre-defined thresholds of RGB color space using ImageJ software (ver 1.52a). Finally, interstitial fibrosis level was expressed as an average ratio of “blue” area against whole tissue area for each animal.

#### Immunoblotting

Each MI-rat heart tissue was homogenized by BioMasher II (320103, Nippi, Tokyo Japan) in T-PER protein extraction reagent (78510, Thermo Fisher Scientific, Waltham, MA) supplemented with protease and phosphatase inhibitor Cocktail (04080-11, Nacalai Tesque, Kyoto, Japan). Then the lysate was centrifuged and cleared extract was denatured by NuPAGE LDS Sample Buffer (NP0007, Thermo Fisher Scientific, Waltham, MA) in non-reduced condition (i.e., without 2-mercaptoethanol). The same amount of whole proteins for each sample were resolved using NuPAGE and transferred to a PVDF membrane by using NuPAGE system and iBlot system (Thermo Fisher Scientific, Waltham, MA), respectively. Then proteins were analyzed using primary antibodies for Col1a1 (R1038, OriGene, Rockville, MD), Col3a1(ab6310, Abcam, Cambridge, UK) and Gapdh (AM4300, Thermo Fisher Scienctific, Waltham, MA), respectively on Amersham Imager 600 (GE Healthcare, Chicago, IL).

#### Isolation of mouse cardiac fibroblasts

According to Zafeiriou et al., briefly 8 to 10 weeks old male C57BL/6 mice were euthanized by CO_2_.^51^ The hearts were dissected and minced to the small pieces of 1–2 mm^3^ with sharp scalpel. The fragments were suspended into the digestion buffer (1U/mL Collagenase Type 2, LS004176, Worthington Biochemicals Corporation, NJ; 1% Trypsin, 15090-046, Thermo Fisher Scientific, MA; HBSS, H6648, Sigma-Aldrich, MO) and incubated at 37°C for 10 min with rotation. The tube was settled for 2 min to make the fragments precipitated, and the supernatant was discarded to remove the blood and debris. Then, to isolate cardiac fibroblasts, another 20 mL of digestion buffer was added to the tube and incubated at 37°C for 10 min with rotation. The tube was settled for 2 min to make the fragments precipitated, and the supernatant that contains cardiac fibroblasts was transferred into a new tube and stored on ice. This procedure was repeated 3 times. After centrifugation (500 g × 5 min), the supernatant was carefully aspirated and the cell pellet was suspended with fibroblast growth medium (FGM-3 Cardiac Fibroblast Growth Medium-3 BulletKit, CC-4526, Lonza, NJ), seeded on a dish coated with 0.5% gelatin (Gelatin Solution, G1393, Sigma-Aldrich, MO) and incubated at 37°C, 5% CO_2_ for 3 to 4 days. The cells grown on the dish was stored in liquid N_2_ as mouse cardiac fibroblasts.

#### hiPSC-derived CM co-culture experiment

4.0x10^6^ human iPSC-derived CMs were seeded into a cell culture insert (Falcon Cell Culture Inserts with 1.0 μm pore membrane, 35312, Corning, NY) precoated with 0.5% gelatin (Gelatin Solution, G1393, Sigma, MO), and then cultured in DMEM/F-12 GlutaMAX supplement (10565018, Thermo Fisher Scientific, MA) with 100 IUml^−1^ penicillin and 100 μg/mL streptomycin (15140122, ThermoFisher Scientific, MA), and incubated for 7 days at 37°C. Next, the hiPSCs-containing inserts were transferred to a 6-well plate confluent with cardiac fibroblasts and incubated in DMEM/F-12 GlutaMAX supplement (10565018, ThermoFisher Scientific, MA) with 100 IUml^−1^ penicillin and 100 μg/mL streptomycin, with or without 10 μM TGF-β inhibitor (SB431542, 1614, TOCRIS, Bristol, UK) for 3 days at 37°C.

#### Real-time PCR

Total RNA was extracted by using Quick-RNA Miniprep Kit (R1055, Zymo Research, Freiberg, Germany), and cDNA was synthesized by using a SuperScript VILO cDNA synthesis kit (11754050, Thermo Fisher Scientific, Waltham, MA). Real-time polymerase chain reaction (PCR) was performed by using PowerUp SYBR Green Master Mix (A25742, Thermo Fisher Scientiric, Waltham, MA) on a StepOnePlus Real-Time PCR system (Applied Biosystems). All measurements were performed in technical triplicate, and the mean value of the three replicates was used for analysis. Details on primer and probe sets can be found in the key resources table. For the quantification of the relative mRNA expression, all data were normalized to Hprt as an internal control and evaluated using the 2^–ΔΔCt^ method. For comparison of the different response to iPSC-CM and TGF-β1 inhibitor treatment, mRNA data are expressed as 2^−ΔΔCt^ by normalization to the control. Statistical analysis was performed by one-way analysis of variance (ANOVA) with the Tukey’s significant difference post hoc test using R program ver.3.6.2 (∗∗∗*p* < 0.001, ∗∗∗∗*p* < 0.0001). For hiPSC-derived CM co-culture experiment, data were analyzed using two-way ANOVA with factors ‘Co-culture (±)’ and ‘SB431542 (±)’, including the interaction term. Post hoc pairwise comparisons were conducted on estimated marginal means with Tukey adjustment.
