# Supplementary material for: Human iPSC cardiomyocyte patch transplantation modifies extracellular matrix and fibroblast behavior after myocardial infarction
Source: iScience. 2026 Mar 11;29(4):115341. doi: 10.1016/j.isci.2026.115341 (PMC13053790; doi:10.1016/j.isci.2026.115341)
Supplement: Document S1. Figures S1–S5 [file mmc1.pdf]

**iScience, Volume *29***

## Supplemental information

# Human iPSC cardiomyocyte patch transplantation modifies extracellular matrix and fibroblast behavior after myocardial infarction

**Kosuke Torigata, Ryohei Matsuura, Fumiya Nagatomo, Moe Thiha, Takao Hikita, Hiroko Iseoka, Hiromitsu Takagi, Uichi Koshimizu, Hiroki Sakakima, Satoshi Izumi, Asuka Hatano, Thomas Braun, Yoshiki Sawa, Shigeru Miyagawa, and Masanori Nakayama**

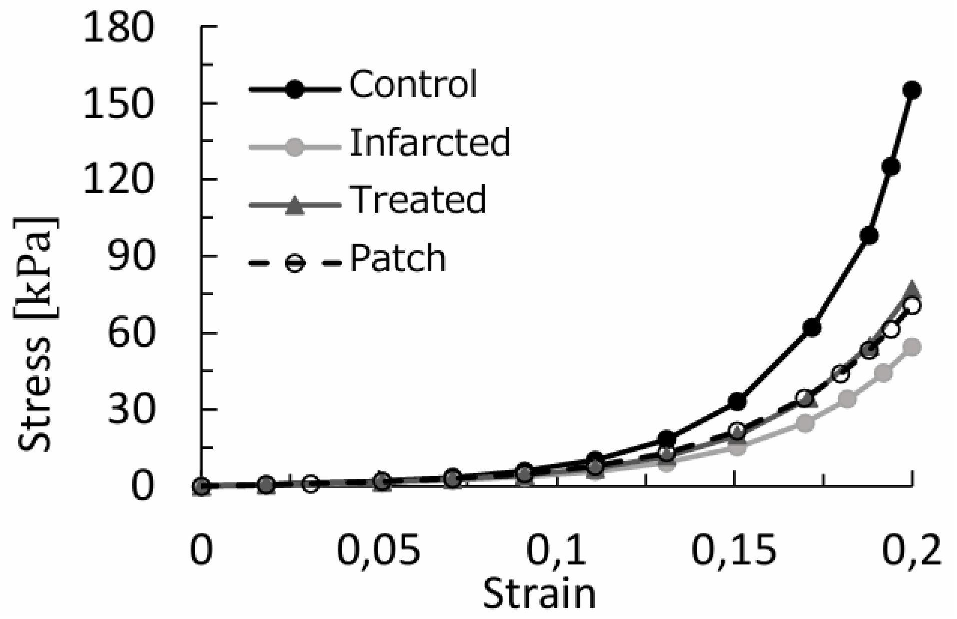


**Figure S1. Passive stress–strain relationship in uniaxial tension tests of material models.**

The axial direction corresponds to the fiber direction for the healthy, infarcted, and implanted models and to the in-sheet direction for the patch model.

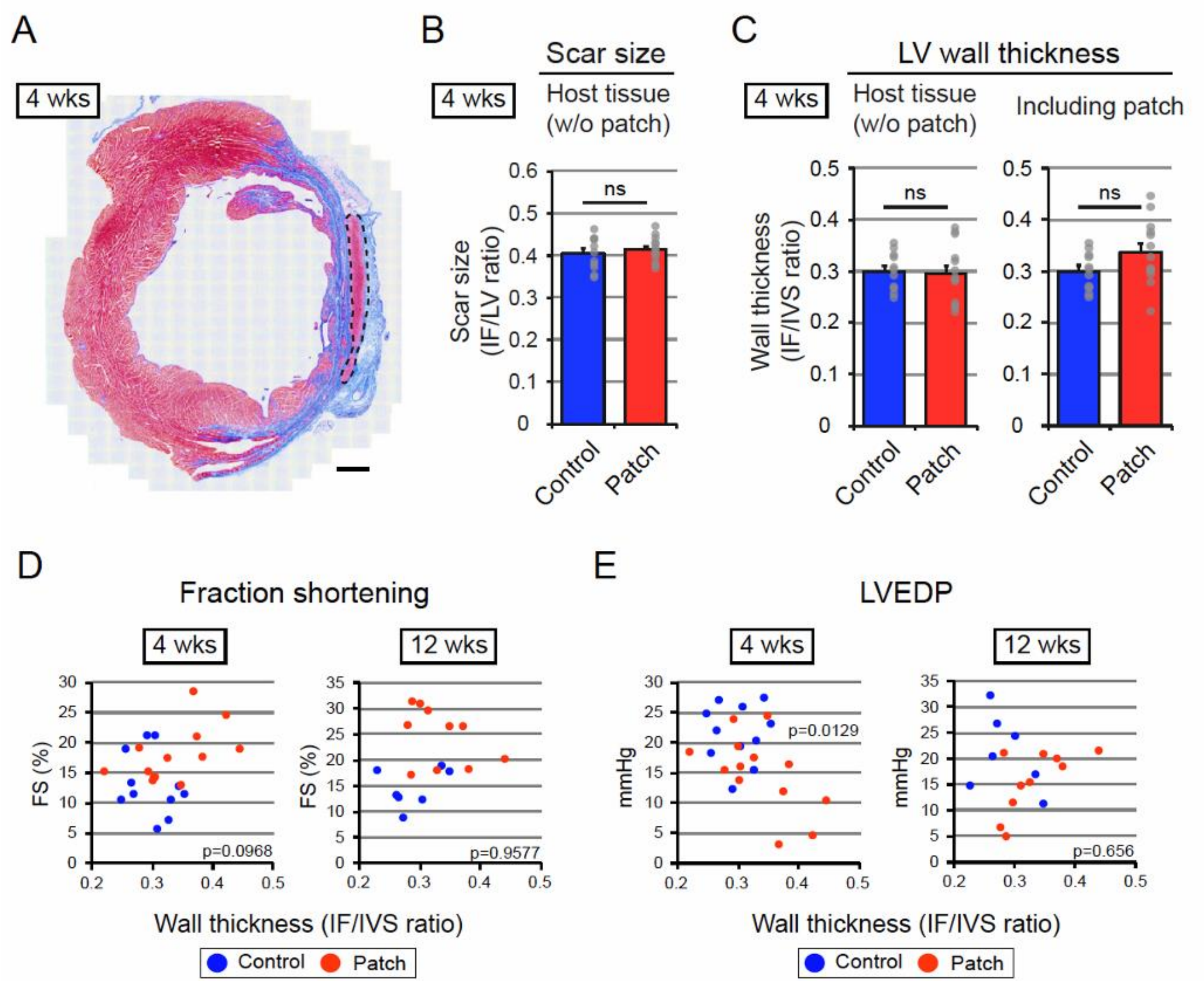


**Figure S2. Wall thickness of the infarct zone does not correlate with cardiac functional recovery.**

(A) Representative image of a hiPSC-derived CM patch–transplanted heart at 4 weeks, visualized by Masson's trichrome staining. Scale bar, 1 mm. (B) Scar size measured 4 weeks after transplantation (n = 11 control; n = 13 transplanted). Data represent mean ± S.E.M. (C) Left ventricular wall thickness with and without transplanted hiPSC-derived CM patch were measured in control and transplanted hearts (n = 11 control; n = 13 transplanted). Data represent mean ± S.E.M. (D) Correlation between wall thickness and fractional shortening at 4 and 12 weeks after transplantation (4 weeks: n = 11 control, n = 13 transplanted; 12 weeks: n = 7 control, n = 10 transplanted). (E) Correlation between wall thickness and LVEDP at 4 and 12 weeks after transplantation (4 weeks: n = 11 control, n = 13 transplanted; 12 weeks: n = 9 control, n = 11 transplanted).

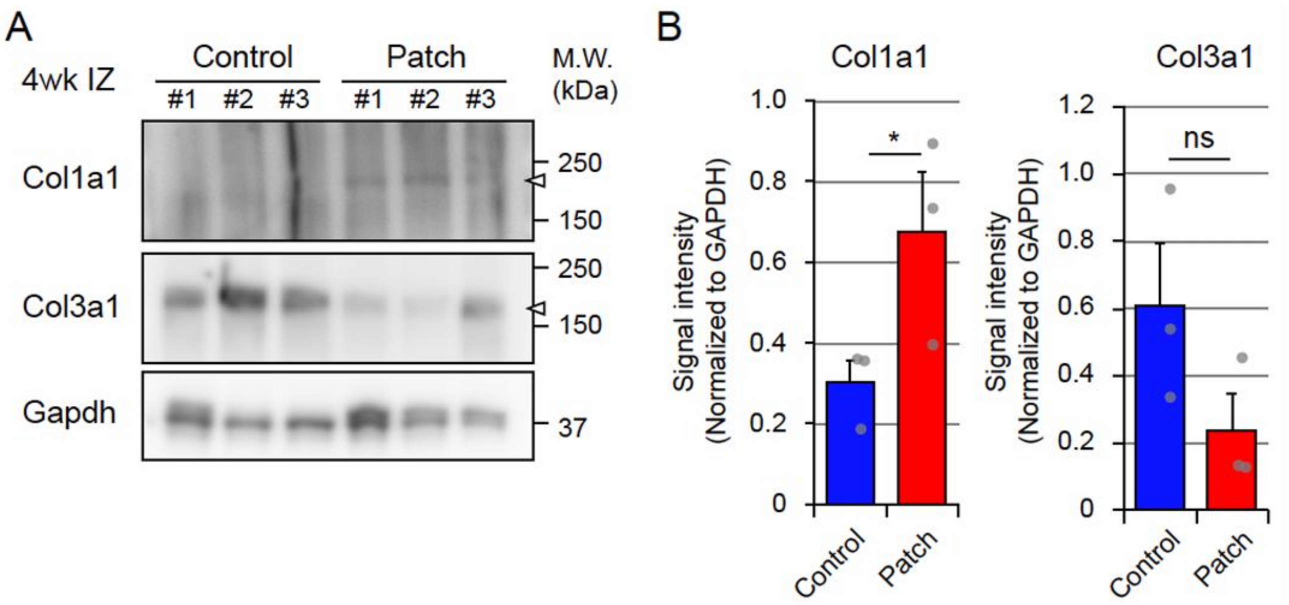


**Figure S3. Native PAGE analysis of collagen type I and type III in infarct zone tissue lysates of control and hiPSC-derived CM transplanted rats.**

(A) Expression of collagen type I and type III in tissue lysates from the infarct zone of control and hiPSC-derived CM–transplanted rats. GAPDH was used as an internal control. Molecular weights are indicated on the right of each panel. (B) Quantification of collagen type I and type III expression levels. Data represent mean ± S.E.M. (n = 3). *$p < 0.05$.

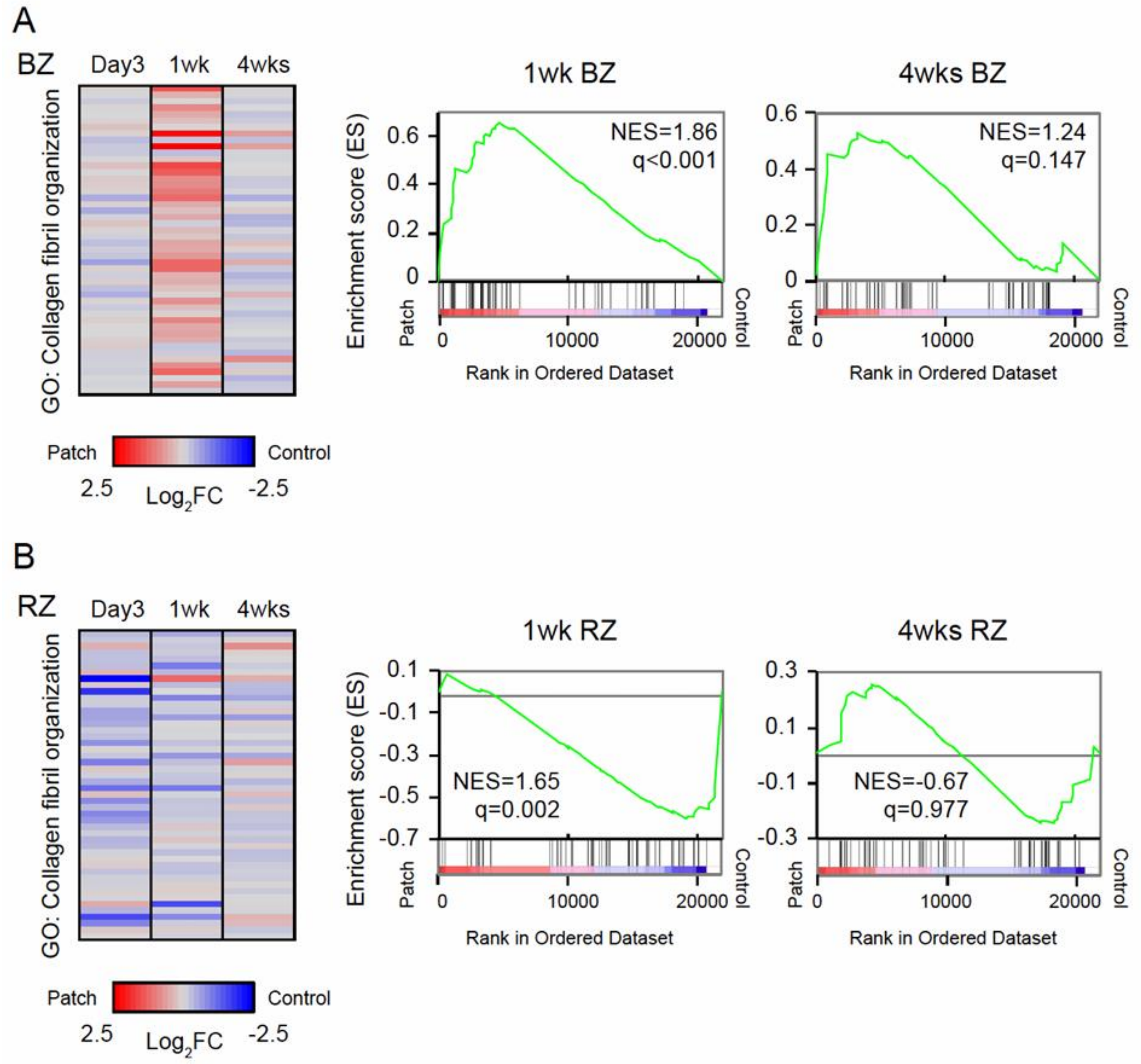


**Figure S4. hiPSC-derived CM patch transplantation alters transcriptional programs related to collagen fibril organization.**

(A, B) Heatmaps of genes in the collagen fibril organization gene set expressed in the border zone (BZ) (A) and remote zone (RZ) (B). Gene set enrichment analysis comparing the patch-transplanted group and control group is shown. Samples were collected 1 week or 4 weeks after transplantation.

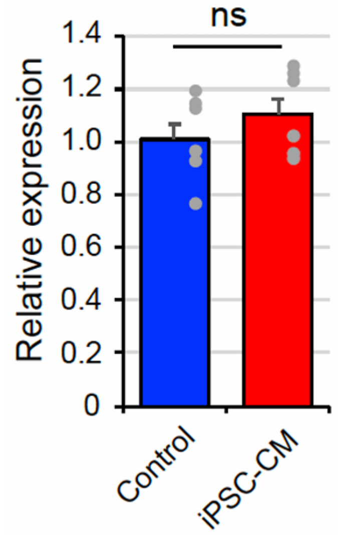


**Figure S5. *Tgfb1* expression in cardiac fibroblasts is not affected by co-culture with hiPSC-derived CMs.**

*Tgfb1* expression in cardiac fibroblasts was not induced by co-culture with hiPSC-derived CMs. Data represent mean ± S.E.M. (n = 6).
